# Identification of genomic regions affecting nitrogen excretion intensity in Brown Swiss dairy cows

**DOI:** 10.1080/10495398.2024.2434097

**Published:** 2024-12-10

**Authors:** Žan Pečnik, Daša Jevšinek Skok

**Affiliations:** a Agricultural Institute of Slovenia, Ljubljana, Slovenia; b University of Ljubljana, Ljubljana, Slovenia

**Keywords:** Association study, dairy cows, functional analysis, genomic regions, nitrogen excretion

## Abstract

Dairy cows with a lower nitrogen excretion intensity (N_exi_) excrete less nitrogen, ammonia (NH_3_) and nitrous oxide (N_2_O), a highly potent greenhouse gas (GHG), per kg of milk produced and therefore represent a lower environmental impact while maintaining food security. To date, the genomics background of N_exi_ is unknown. Here we performed a genetic association study, overlap analysis and functional enrichment analysis for N_exi_ in 875 genotyped dairy cows with 2,147 lactations from 200 herds. We identified 1456 single nucleotide polymorphisms (SNPs) that significantly affect N_exi_. We found 140 SNPs overlapping with 148 protein-coding genes. The *MAN1A1* gene is a strong candidate gene for N_exi_. Genotype CC of rs42786248, the most significantly associated SNP located in the *MAN1A1* gene, had higher N_exi_ than genotypes AA (*p* < 0.001) and AC (*p* < 0.001). We identified 33 genes involved in biological processes related to nitrogen metabolism. Our results form the basis for further research on the genomics background of N_exi_. The identified SNPs serve as potential targets for selective breeding programs, aimed at reducing N_exi_ and associated NH_3_ and N_2_O emissions in cattle production, thus contributing to more environmentally sustainable milk production.

## Graphical abstract

**Figure:**
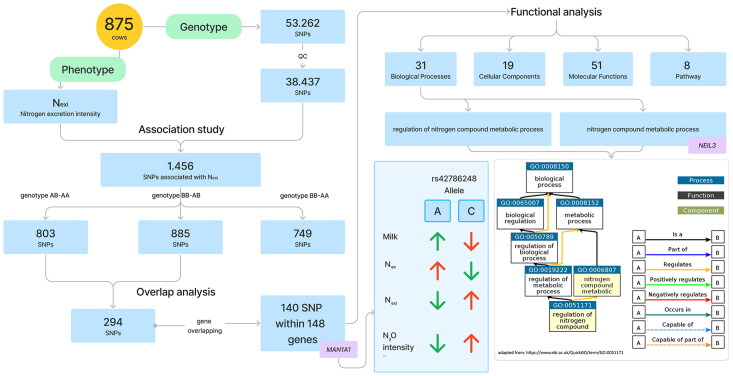

## 1. Introduction

The excretion of various nitrogen (N) compounds from dairy cattle leads to environmental problems. As recently reported, the livestock sector urgently needs a global initiative to tackle N pollution while supporting food security.^1^ Cattle excrete nitrogen through feces and urea, which is further transformed into ammonia (NH_3_), and nitrous oxide (N_2_O), a highly potent greenhouse gas. In the latest IPCC assessment report^2^ the global warming potential (GWP_100_) for N_2_O was estimated at 273 ± 130, which is much higher compared to methane (CH_4_), where the GWP_100_ for non-fossil CH_4_ is 27.0 ± 11. In addition, CH_4_ remains in the atmosphere for 11.8 ± 1.8 years, while N_2_O accumulates for 109 ± 10 years^2^ and can be defined as long-lived. The livestock sector emits one-third of human-induced N emissions^1^ and N_2_O mitigation measures in dairy herds with declining or constant emissions will be more important than CH_4_ mitigation measures in the future as the warming effect of methane is often overestimated.^3^

N_2_O emissions originate from urine and manure nitrogen excretion (N_ex_) in processes of nitrification and denitrification. Most of the N-rich compounds are excreted in urine and are products of complex ruminant N-metabolism, where crude protein is digested by microorganisms to peptides, amino acids and NH_3_. Kohn et al.^4^ have reported a close relationship between the level of crude protein in the feed ration and the amount of N released into the environment. In this way, 75–85% of the excess proteins consumed by a dairy cow are excreted in urine, feces and milk.^4^^,^^5^ To obtain information on total N_ex_, measurements are laborious, expensive and impractical at large scales; therefore, models are needed to predict N excreted in urine and feces. Different models enable quantification of urinary, feces or total N_ex_ and different phenotypes could be used.^6^ The total estimated N_ex_ is subsequently used to calculate N_2_O emission at individual animal levels.

Strategies to reduce N_ex_ indirectly mitigate N_2_O emissions.^7^ Technical options for mitigation of N_ex_ and N_2_O emissions were clearly defined,^8^^,^^9^ and breeding was recently recognized as a valuable contribution to the whole set of mitigation strategies that could be applied to achieve the temperature goals for 2050 set by the EU.^10^ Furthermore, farmers are actively seeking highly efficient cows concerning N_ex_ and milk production.^11^ Efficient cows are those that, for the same amount of milk production, excrete lower amounts of N in both urine and feces, i.e., have lower nitrogen excretion intensity (N_exi_). N_exi_ and N_2_O emission intensity are calculated by dividing the sum of N_ex_ and total N_2_O emissions, respectively, by the total milk production over the selected timescale. Genetic selection for total N_ex_ could be conflicting with other desirable traits in breeding programs, such as total milk production. This is because cows with higher milk production tend to excrete more nitrogen^4^ and selecting for such animals may result in lower overall milk production. Hence, we are interested in the genomics background of N_exi._ Moreover, determining N_ex_ and N_exi_ is important for assessing and managing N_2_O emissions in subsequent stages of manure management. If we identify genomic regions associated with N_exi_, we can indirectly mitigate N_2_O emission intensity.

Mitigation potential for N_2_O with genetic selection is uncertain due to limited research or lack of data.^12^ Some genetic studies aimed to reveal the genetic background for N_ex_-related traits. Honerlagen et al.^13^ identified genomic regions and candidate genes influencing nitrogen-excretory metabolites in the urine of Holstein-Friesians. These genes were *ITPR2* and *MYBPC1* (BTA 5), *STIM2* (BTA 6), *SGCD* (BTA 7), *SLC6A2* (BTA 18), *TMCC2* and *MFSD4A* (BTA 16). Chen et al.^14^ identified 16 candidate genes associated with NEI, a trait that includes N intake, milk true protein N and milk urea N yield. These genes were mainly expressed in the milk cell, mammary and liver tissues, with several of them being involved in N metabolism. Examples include *CA1*, *CA2*, *CA3*, *CA8*, *CA13*, *LOC784254* and *LOC100847874*, all of which are located on BTA14. Recently, Kong et al.^15^ reported that the length of cilia in kidney cells is associated with urine concentration in mice kidneys. The length of cilia is linked to the *CFAP77* gene, which was recently also associated with urea N content in Holstein cattle.^16^ Pegolo et al.^17^ identified genomic regions associated with milk N fractions and highly interconnected genes in them. It was shown recently that high genetic merit Holstein cows in Northern Ireland utilize feed N for milk production more efficiently than lower genetic merit cows.^18^ Consequently, modern dairy cows can partition more ingested N into milk and excrete less N in feces and urine, per kilogram of standard milk.^18^ Manzanilla-Pech et al.^19^ conducted a genomic study on CH_4_ emission intensity, expressed as concentration and production. They linked three SNPs to both traits, namely Hapmap59221-rs29014908 and Hapmap44201-BTA-114510, both on BTA4, and Hapmap52436-rs29009653 on BTA6. However, to the best of our knowledge, there have been no studies investigating the genomic background of N_exi_.

Our study aims to fill the existing knowledge gap of the genomic background of N_exi_ in dairy cows. We focus on (1) the identification of single nucleotide polymorphisms (SNPs), candidate genes and gene ontologies associated with N_exi_; (2) conducting association analysis, overlap analysis and functional enrichment analysis; (3) estimation of differences in N_2_O emissions between genotypes of most significant SNP associated with N_exi_. Our ultimate objective is to establish a breeding strategy aimed at minimising N_exi_, thereby making a significant contribution to the reduction of N_2_O emissions while sustaining milk production.

## 2. Material and methods

### 2.1. Dataset

We used the data of 875 genotyped Brown Swiss dairy cows from the Slovenian cattle population, with phenotypes collected in the Central cattle database^20^ of the Agricultural Institute of Slovenia. Briefly, animal ID, calving ID, calving date, urea content and milk production in full lactation were recorded during the regular monthly milk recordings. As this was an *in silico* study, approval from the animal care and use committee approval was not required. The inter-calving period was calculated as the sum of days between two consecutive calvings. We used 2,147 lactation records collected in 200 herds from 2008 and 2022.

### 2.2. Phenotype determination

We estimated N_ex_ from milk recording data for all 875 genotyped dairy cows. To calculate N_ex_ (expressed in kg N per animal per year), we modified the following equation:^21^ 129.9 + 0.0089 ∗ (milk − 7,744) + 1.7 ∗ (urea − 26), where ‘milk’ represents milk production (in kg per animal per year) and ‘urea’ represents urea content (in mg/dl). The values 0.882 for slurry and 0.800 for solid manure in the^21^ equations were redundant for our purpose. Our goal was to estimate the phenotype of the animals, so the volatilisation factors 0.882 or 0.800 were not included in our estimation. Furthermore, our milk recording data provided information on milk production throughout the lactation period (milk_f). Therefore, to obtain annual values, we adjusted to account for the inter-calving period, resulting in the final N_ex_ equation:

N_ex_ = 129.9 + 0.0089 ∗ (milk_f ∗(365/IP) − 7,744) + 1.7 ∗ (urea − 26),where IP represents the inter-calving period in days.

To assess the N_exi_, we calculated N_exi_ as the ratio of N_ex_ to milk yield during the full lactation period, adjusted for the inter-calving period (IP), using the formula:

$$N_{\text{exi}}={\text{ N}}_{\text{ex}}/\left(\text{milk}\_\text{f }*\left(365/\text{IP}\right)\right)$$

This approach allowed us to provide a comprehensive estimation of N_exi_ while considering the variables of milk production, urea content and the inter-calving period.

### 2.3. Ammonia and nitrous oxide emissions

We assessed the emissions of NH_3_ and N_2_O from housing and storage areas, respectively, based on N_ex_ to determine the total emissions per kilogram of milk and about candidate genes significantly associated with N_exi_. We utilized the methodologies of the European Environment Agency^22^ and the Intergovernmental Penal for Climate Change.^23^^,^^24^ Due to the absence of data on farming practices, it was assumed that excreta were collected in the form of slurry without bedding residues, animals did not graze and no emission reduction measures were implemented on the farms. Since all excreta are collected in housing facilities, according to the EMEP/EEA^22^ guidelines, the annual N excreted in-house (m_hous_N_) is represented by the equation m_hous_N_ = N_ex_ holds. Additionally, given that all excreta are collected in slurry and housing facilities (m_hous_slurry_TAN_, where TAN stands for total ammonia nitrogen), the equation m_hous_slurry_TAN_ = m_hous_TAN_ holds. First, we calculated total ammonia nitrogen (TAN) as the amount of TAN deposited during housing: m_hous_slurry_TAN_ = x_TAN_ ∗ m_hous_TAN_. Next, we estimated ammonia emissions (NH_3_–N) as kg NH_3_-N by multiplying m_hous_slurry_TAN_ by the emission factor EF_housing_ = 0.24.^22^ Next, we calculated TAN (entering store = TAN transferred from housing to the storage areas) as the difference between ammonia emissions and TAN deposited during housing ammonia (NH_3_–N) emissions by multiplying TAN (entering store) by the emission factor EF_storage_ = 0.25.^22^ Next, we assessed the indirect emissions of N_2_O from NH_3_ emissions in housing by considering that 1% of the emissions are converted into N_2_O as N_2_O–N,^23^ i.e., we multiplied indirect NH_3_ emissions by 0.01. To estimate the indirect emissions of N_2_O from NH_3_ emissions in storage, we multiplied TAN (entering the store) by 0.02.^22^ We then considered converting N_2_O-N to N_2_O by multiplying emissions by conversion factor 44/28 for both direct and indirect emissions. Finally, for conversion into CO_2_ equivalents, a GWP_100_ factor of 265^25^ was applied.

### 2.4. Association study

To conduct an association study, we combined genotypic datasets with phenotype data. A total of 875 cows were genotyped using SNP microarray ICBF International Dairy and Beef v3 (ICBF, Ireland) for a total of 53,262 SNP markers. Polymorphisms were annotated by SNPChimp v3.^26^ Polymorphisms without a mapped rs ID or commercial ID in the current genome assembly were assigned placeholder coordinates (chromosome 99, position 0) to differentiate them from SNPs originally located on chromosome 0 in platform files. Quality control (QC) of the genotype data was done using PLINK.^27^ For the details of QCs in this study, we refer to Anderson et al.^28^ Finally, a total of 38,427 SNPs were retained.

The association analysis, using one SNP at a time, was performed with a linear mixed model using the ‘*lme4*’ package in R software.^29^ The single SNP regression analysis was performed using the following statistical model:

(1) $$y_{\text{ijklmn}}=\;\mu +P_{i}+G_{j}+S_{k}+b\left(x_{\text{ijklm}}-\;\bar{x}\right)+c_{l}+h_{m}+e_{\text{ijklmn}}\;$$

where *y_ijklmn_* = N_exi_ (g/kg milk), *µ* = overall mean, *P_i_* = parity (*i* = 1–3), *G_j_* = genotype (*j* = 1–3), *S_k_* = season (*k* = 1–4), *x_ijklm_* = age at calving, *c*_l_ = random additive effect for sire l (*l* = 1, 2, …, 227), *h_m_* = random effect for herd *m* (*m* = 1, 2, …, 200) and *e_ijklmn_* = a random residual.

To account for multiple comparisons and control the false discovery rate, we applied the false discovery rate (FDR) correction method. The FDR correction was used to adjust the *p*-values obtained from the mixed model analysis, ensuring a more stringent control of Type I errors. The significance threshold after FDR correction was set at 0.01.

### 2.5. Genomic overlap analysis

Genomic overlap analysis was performed comparing the location of SNPs associated with N_exi_, and protein-coding genes. Genes were obtained from Ensembl Biomart Release 112, while gene nomenclature was based on the HUGO Gene Nomenclature Guidelines (http://www.genenames.org). Throughout the text, genomic positions of SNPs and genes were based on the ARS-UCD1.3 Bovine Genome Assembly. Views (graphical overview of the chromosomal locations) were constructed using the visualisation tool Flash GViewer (http://gmod.org/wiki/Flashgviewer/) developed by the GMOD project.

### 2.6. Functional analysis

Functional analysis for a set of SNPs associated with N_exi_ was conducted by the public web server GeneTrail3, where the analysis for 12 model organisms, including cattle can be done. The statistical significance threshold was set to *p* < 0.05. Over-representation analysis (ORA) as an enrichment algorithm with the FDR adjustment method was used to determine the number of statistically significant categories.^30^ We included the Ontology and Phenotype section (GO—Biological Process, GO—Cellular Component, GO—Molecular Function), Pathways (KEGG-Pathways, Reactome—Pathways and WikiPathways), Genomic positions from NCBI Assembly and analysis of protein families based on Pfam database.

## 3. Results and discussion

### 3.1. Phenotypes: nitrogen excretion and nitrogen excretion intensity in dairy cows

The variability in milk production within the studied Slovenian population of Brown Swiss dairy cows was substantial. On average, dairy cows produced 7,337 kg of milk throughout the entire lactation period, with differences between the most and least productive exceeding fourfold (Table 1). The shortest inter-calving interval was 291 days, whereas the longest one was nearly two and a half years. The urea content was, on average, optimal, although certain individuals showed values that were either too low or too high. On average, the cows excreted 105.6 kg of N annually, translating to 17.52 g of N_exi_ and 314.04 N_2_O emissions expressed in kilograms of CO_2_ equivalents.

**Table 1.** Descriptive statistics for phenotype traits of 875 Brown Swiss dairy cows.

Trait^a^	Mean	Standard deviation	Minimum	Maximum
Milk production in a year (kg)	6,206	1,641	2,724	12,539
Milk production in full lactation (kg)	7,337	2,343	3,063	16,637
Inter-calving period (day)	436	100	291	912
Milk urea (mg/100 ml)	19.77	5.22	6.00	37.58
N_ex_ (kg/year)	105.6	18.9	61.0	190.7
N_exi_ (g/kg milk)	17.52	2.59	12.40	28.21
NH_3_ emissions (kg/year)	15.210	2.719	8.778	27.464
N_2_O direct housing emissions (kg CO_2_ eq/year)	63.34	11.32	36.56	114.37
N_2_O direct storage emissions (kg CO_2_ eq/year)	200.57	35.85	115.76	362.16
N_2_O indirect storage emissions (kg CO_2_ eq/year)	50.14	8.96	28.94	93.94
N_2_O emissions (kg CO_2_ eq/year)	314.04	56.14	181.26	588.39
N_2_O emission intensity (g CO_2_ eq/kg milk)	52.10	7.70	36.87	96.25

^a^N_ex_: nitrogen excretion; N_exi_: nitrogen excretion intensity; NH_3_: ammonia; N_2_O: nitrous oxide; SD: standard deviation.

N_ex_ and N_exi_ are dependent on milk production. N_ex_ was 20 kg lower and N_exi_ higher 5.5 kg per kg of milk than values from a meta-analysis of 86 nitrogen balance trials in ref.^31^ They found that the average nitrogen content in feces and urine was 186 g/day and 192 g/day. When we recalculate these values and consider the average milk production of 31.4 kg of milk per day, we conclude that N_ex_ in their study was 138 kg over an average year of 365.25 days, which amounts to a N_exi_ of 12 g/kg milk. Therefore, N_ex_ was 20 kg higher, while N_exi_ was approximately 5.5 g/kg of milk lower. This is understandable because the animals had a significantly higher daily milk yield, and the excreted nitrogen was distributed over the total milk production.

### 3.2. Association analysis and overlap analysis

**Figure 1.**
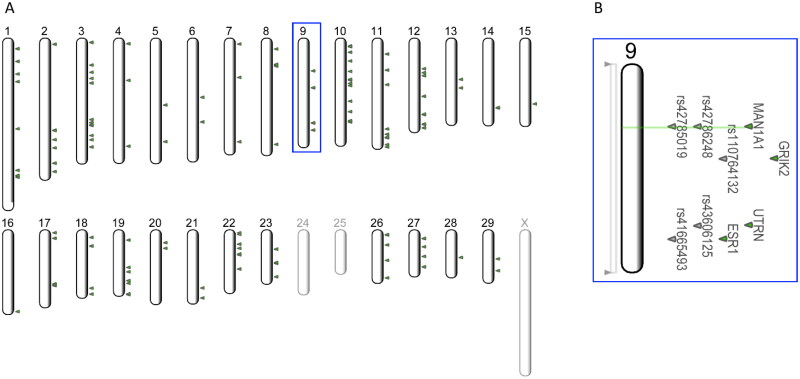
(A) Bovine chromosomes with 140 SNPs associated with nitrogen excretion intensity (N_exi_), overlapping 148 protein-coding genes. (B) Enlargement of chromosome 9 showing an overlap between gene *MAN1A1* and two SNPs (rs42786248 and rs42785019) associated with N_exi_.

To identify SNPs associated with N_exi_ we carried out an association study on the cows with calculated N_exi_. We performed an association study between N_exi_ data and 38,437 SNPs. While considering effects of parity, season, age at calving, random additive effect for sire, random effect for herd and residual in the mixed model, we found 1,456 SNPs associated with N_exi_ (adjusted *p*-value < 0.01) (Supplementary Table S1). Genotype contrasts were computed to more accurately assess the influence of an individual SNP. We discerned distinctions between heterozygous (AB) and homozygous individuals (AA, coded as 0 for homozygous reference, and BB, coded as 2 for homozygous alternative) by analysing the estimated values of N_exi_. We found 803 SNPs with a significant difference between genotypes AB–AA, and 885 SNPs between genotypes BB and AB, while between genotypes BB and AA 749 SNPs were found. There were 294 SNPs with differences between heterozygous and homozygous genotypes. We overlapped the genomics regions of this set of 294 SNPs with genomics regions of protein-coding genes (Figure 1).

Most of the 294 SNPs are intergenic variants (132), followed by SNPs located within introns of protein-coding genes (123), 21 SNPs are located upstream of the gene region and 19 downstream of the gene region. Seven SNPs are located within coding regions of genes, five (rs109623009, rs109816907, rs110710907, rs110862887 and rs43712268) of them are missense variants and two of them are synonymous variants rs110501981, and rs43498404 within genes *RTL1*, and *USHBP1*, respectively (Supplementary Table S1). Notably, SNP rs42786248 stands out as the most significant SNP, exhibiting a strong association with N_exi_, as evidenced by the lowest adjusted p-value (1.412243e-07). The *MAN1A1* gene is involved in the protein N-glycosylation processing phase (mammalian) and is overexpressed in the liver.^32^^,^^33^

The 140 SNPs are distributed across 148 different genes (Supplementary Table S1). The ten most significantly associated SNPs (and their overlapping genes) with N_exi_ are rs42786248 (*MAN1A1*), rs42805044 (*ADAMTSL1*), rs110905653 (*ITFG1*), rs110905653 (*PHKB*), rs109746439 (*RNF44*), rs109040251 (*PDE8B*), rs42436662 (*NEIL3*), rs109645596 (*RXRA*), rs29013388 (*NEIL3*) and rs42570718 (*ENSBTAG00000066084*). The SNP rs110905653 overlaps with two genes, namely *ITFG1* and *PHKB*. Moreover, two SNPs rs42436662 and rs29013388 overlap with the *NEIL3* gene*. NEIL3* was previously associated with renal clear cell carcinoma^34^^,^^35^ and hepatocellular carcinoma^35^ in humans. The nuclear factor 1 A (*NFIA*) gene was involved as a transcription factor that plays a role in the development of the liver in humans.^36^ To obtain a better understanding of the functions of genes associated with N_exi_, we performed a functional enrichment analysis.

### 3.3. Functional enrichment analysis: biological processes, cellular components, molecular functions and pathways associated with nitrogen excretion intensity

**Figure 2.**
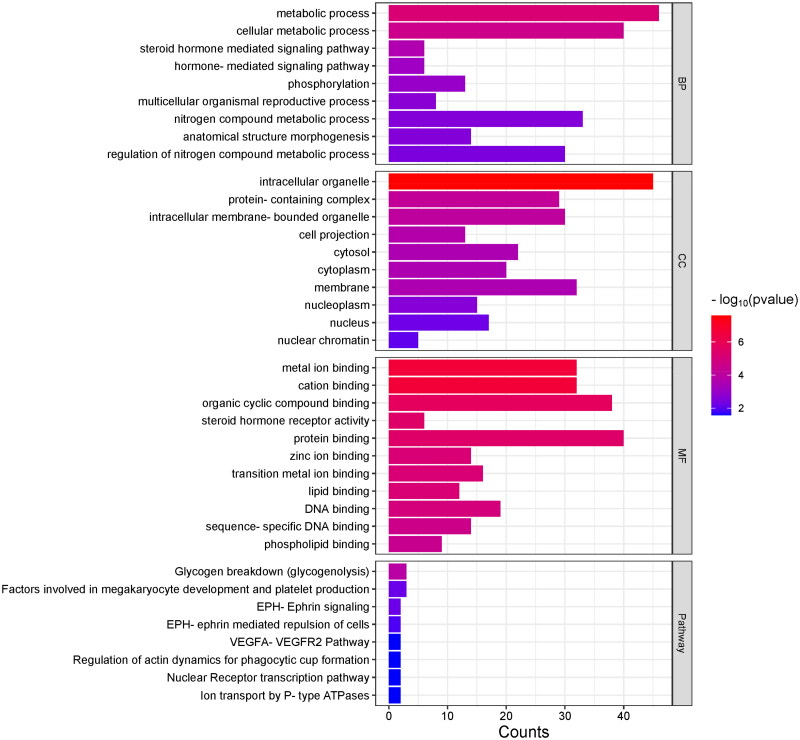
Ranking of the top 10 biological processes (BP), cellular components (CC) and molecular functions (MF) based on *p*-value, identified by functional analysis. Gene ontologies in individual sets are ordered by P/FDR from smaller to larger values.

**Figure 3.**
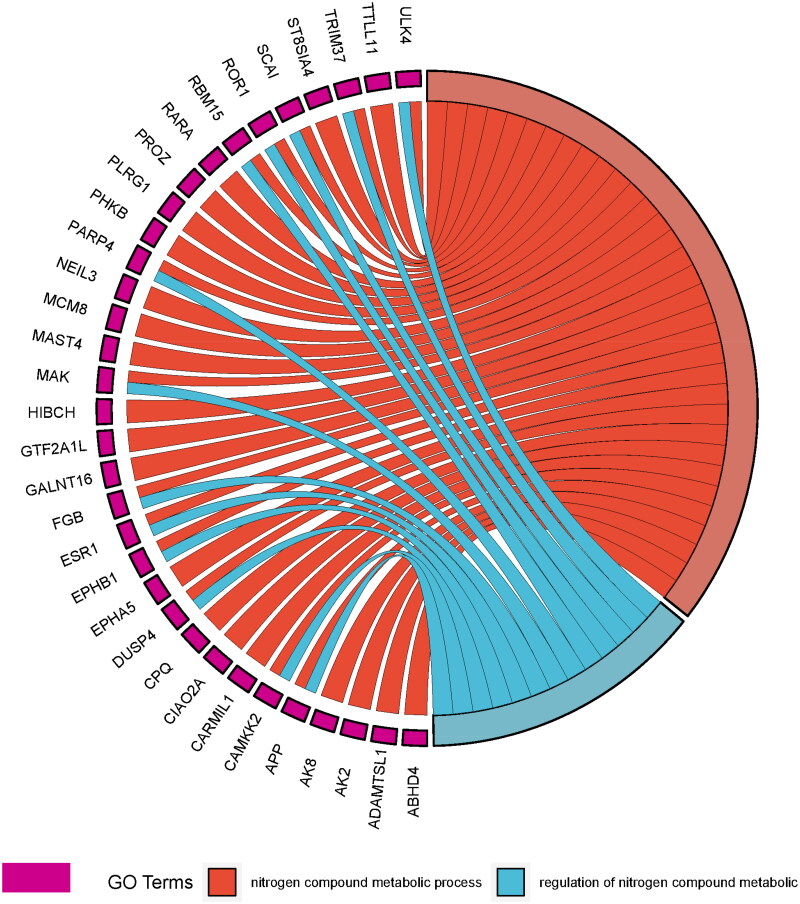
Relationship between two GO terms associated with nitrogen term and genes included in these biological processes.

The analysis of gene ontology (GO) and biological pathway was performed for 148 genes resulting from the overlap analysis. Our gene sets were compared with the GeneTrail3 GO knowledgebase, which contains 875 biological processes (BP), 124 cellular components (CC), 193 molecular functions (MF) and 23 Reactome Pathways. We identified 31 significant BP, 19 CC, 51 MF and 8 Reactome Pathways involving the set of 148 genes (Supplementary Figure 1). The top 10 gene ontologies within individual GO classes were selected based on the 10 lowest *p*-values resulting from our functional analysis (Figure 2). Additionally, Figure 3 showcases both significant biological pathways derived from the Reactome database.

#### 3.3.1. Biological processes

In total, 97 genes are involved in at least one significant biological process. We identified 31 significant biological processes in which our gene set played a crucial role. The ten most noteworthy biological processes were as follows: developmental process, metabolic process, cellular metabolic process, steroid hormone-mediated signalling pathway, hormone-mediated signalling pathway, phosphorylation, multicellular organismal reproductive process, anatomical structure morphogenesis, nitrogen compound metabolic process, and regulation of nitrogen compound metabolic process. Two significant processes were associated with nitrogen compounds. In addition, the biological process called cellular response to peptide was also involved in response to nitrogen compounds. We found 33 unique genes in processes associated with nitrogen. In the biological process ‘regulation of nitrogen compound metabolic process’, we found 13 genes, while the process called ‘nitrogen compound metabolic process’ included 33 genes. Genes associated with the biological process ‘nitrogen compound metabolic process’ are *ABHD4*, *ADAMTSL1*, *AK2*, *AK8*, *APP*, *CAMKK2*, *CARMIL1*, *CIAO2A*, *CPQ*, *DUSP4*, *EPHA5*, *EPHB1*, *ESR1*, *FGB*, *GALNT16*, *GTF2A1L*, *HIBCH*, *MAK*, *MAST4*, *MCM8*, *NEIL3*, *PARP4*, *PHKB*, *PLRG1*, *PROZ*, *RARA*, *RBM15*, *ROR1*, *SCAI*, *ST8SIA4*, *TRIM37*, *TTLL11* and *ULK4*. Additionally, the genes *APP*, *CAMKK2*, *DUSP4*, *EPHB1*, *ESR1*, *FGB*, *MAK*, *PARP4*, *RBM15*, *ROR1*, *SCAI*, *TRIM37* and *ULK4* are associated with both nitrogen compound metabolic process and regulation of nitrogen compound metabolic process (Figure 3).

#### 3.3.2. Cellular components

Nineteen out of 124 cellular components were significant in our gene enrichment analysis with 98 genes from the list of 148 genes. Significant cellular components were intracellular organelle, protein-containing complex, intracellular membrane-bounded organelle, cell projection, cytoplasm, cytosol, membrane, nucleoplasm, nucleus, nuclear chromatin, filopodium, nuclear speck, neuromuscular junction, lamellipodium, transcription factor complex, cytoplasmic vesicle, intracellular vesicle, neuron projection and chromatin. The most significant cellular component called ‘intracellular organelle’ contained 45 genes.

We found 29 genes (*APP*, *BPTF*, *CARMIL1*, *CIAO2A*, *CRYBG3*, *ELP3*, *ESR1*, *F2RL2*, FGB, IKZF1, *KNTC1*, *LTBP1*, *MCM8*, *MRPL47*, *NR5A2*, NR6A1, *PBX1*, *PLRG1*, *RBM15*, *ROR1*, *RUNX2*, *RXRA*, *SGIP1*, *SNX1*, *SPATS2L*, TFDP1, *TRIM37*, *UTRN* and *ZFYVE9*) that are involved in cellular component functional pathway protein-containing complex. Some of these genes have been studied in liver and kidney function. The *APP* gene is highly expressed in the liver and is involved in liver metabolism.^37^^,^^38^ The *ESR1* gene encodes for oestrogen receptor alpha, which is expressed in the liver and has been shown to play a role in liver regeneration.^39^ The *KNTC1* is upregulated in liver cancer.^40^ The *LTBP1* has also been found to be involved in the formation of extracellular matrix, which is important for liver development and function.^41^

#### 3.3.3. Molecular functions

In the realm of molecular functions, we found 89 genes involved in 51 significant molecular functions. The top ten MF molecular functions are cation binding, metal ion binding, organic cyclic compound binding, protein binding, steroid hormone receptor activity, lipid binding, transition metal ion binding, zinc ion binding, DNA binding and sequence-specific DNA binding. The *RARA* gene is associated with the most molecular functions, with as many as 40, such as cation binding, protein binding, protein kinase binding, signalling receptor binding, transcription coregulator activity, etc.

#### 3.3.4. Pathway analysis

In the analysis of Reactome pathways, we found eight significant pathways, namely: Glycogen breakdown (glycogenolysis), EPH-Ephrin signalling, Factors involved in megakaryocyte development and platelet production, EPH-ephrin mediated repulsion of cells, Ion transport by P-type ATPases, Nuclear Receptor transcription pathway, Regulation of actin dynamics for phagocytic cup formation, and VEGFA-VEGFR2 Pathway. In glycogenolysis, genes *AGL, PGM2* and *PYGL* are involved, while genes *KNTC1* and *SCAI* play an important role in RHO GTPases Activate Formins biological pathway.

Additionally, we conducted an in-depth enrichment analysis of two genes *MAN1A1* and *NEIL3* (Table 2). Polymorphisms located within those genes were shown as the most significant in our association study. Gene *MAN1A1* is involved in six cellular components and four molecular functions, while gene *NEIL3* is involved in 11 biological processes, five cellular components and 11 molecular functions. Interestingly, gene *NEIL3* is contained in the nitrogen compound metabolic process.

**Table 2.** Gene ontology terms for most promising candidate genes *MAN1A1* and *NEIL3* detected for N_exi_ in dairy cows.

Gene	Contained in	Gene ontology
*MAN1A1*	Intracellular organelle	Cellular Components
	Cytosol	
	Cellular component	
	Intracellular membrane-bounded organelle	
	Integral component of membrane	
	Intrinsic component of membrane	
	Metal ion binding	Molecular Functions
	Molecular function	
	Calcium ion binding	
	Cation binding	
*NEIL3*	DNA metabolic process	Biological Processes
	Cellular aromatic compound metabolic process	
	Cellular metabolic process	
	Nitrogen compound metabolic process	
	Response to stimulus	
	Biological process	
	Heterocycle metabolic process	
	Metabolic process	
	Nucleobase-containing compound metabolic process	
	DNA repair	
	Cellular response to DNA damage stimulus	
	Intracellular organelle	Cellular Components
	Nucleoplasm	
	Cellular component	
	Intracellular membrane-bounded organelle	
	Nucleus	
	Transition metal ion binding	Molecular Functions
	Organic cyclic compound binding	
	Metal ion binding	
	Double-stranded DNA binding	
	Catalytic activity	
	Hydrolase activity	
	Nucleic acid binding	
	Molecular function	
	DNA binding	
	Cation binding	
	Zinc ion binding	

### 3.4. Effect of most significant SNP associated with N_exi_ on NH_3_ and N_2_O emissions

**Figure 4.**
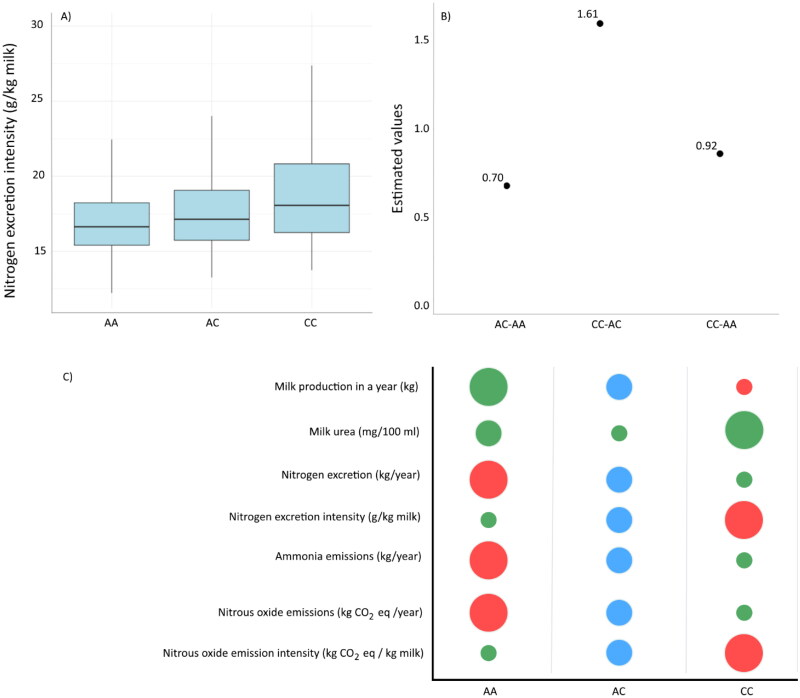
Effect of the rs42786248 SNP on nitrogen excretion intensity. Reference allele A, alternative allele C. (A) Distribution of raw data for N_exi_ among genotypes for SNP rs42786248; (B) estimated differences for N_exi_ between genotypes (AA, AC and CC); (C) Bubble graph illustrating the influence of SNP rs42786248 genotypes (AA, AC and CC) on seven studied traits. Bubble size corresponds to trait value, with larger bubbles indicating higher values. Colours represent desirability: red for undesirability, green for high desirability and blue for moderate desirability.

The most significant SNP from our association study is rs42786248, located within the intron of the *MAN1A1* gene on chromosome BTA9 (Figure 4). We calculated N_ex_, N_exi_, NH_3_ emissions, N_2_O emissions and N_2_O emission intensity separately for cows with each rs42786248 genotype. Cows with genotype CC were significantly less efficient than animals with genotypes AA and AC, as the N_exi_ in genotype CC was 1.49 g/kg milk higher than in genotype AA (*p* < 0.001) and 1.03 g/kg milk in genotype AC (*p* < 0.001), respectively (Table 3). Cows with the genotype AA had a lower N_exi_ of 0.46 g/kg milk compared to individuals with the AC genotype, with statistical significance (*p* < 0.001). Cows with the lowest N_exi_ had the highest amount of milk, N_ex_ and total emissions, but on the other hand, also the lowest N_2_O emission intensity. Cows with genotype CC had 4.43 g CO_2_ equivalent per kg milk lower N_2_O emission intensity than animals with genotype AA (Table 3). Further analysis should focus on confirming the relevance of these candidate genes in dairy cow populations and ensuring their suitability for marker-assisted selection in the context of N_exi_-related traits. This should include assessing their functional role, evaluating their association with important phenotypic traits and verifying their consistency across different population subgroups.

**Table 3.** Phenotypic characteristics of dairy cows with rs42786248 SNP within *MAN1A1* gene.

	Genotype		
	AA	AC	CC
Milk production in a year (kg)	6,375	6,141	5,628
Milk urea (mg/100 ml)	19.76	19.71	20.1
N_ex_ (kg/year)	107.1	104.9	101.0
N_exi_ (g/kg milk)	17.19	17.65	18.68
NH_3_ emissions (kg/year)	64.23	62.93	60.58
N_2_O direct housing emissions (kg CO_2_ eq/year)	203.38	199.28	191.85
N_2_O direct storage emissions (kg CO_2_ eq/year)	50.85	49.82	47.96
N_2_O indirect storage emissions (kg CO_2_ eq/year)	318.45	312.3	300.39
N_2_O emissions (g CO_2_ eq/kg milk)	51.10	52.48	55.55

N_ex_: nitrogen excretion; N_exi_: nitrogen excretion intensity; NH_3_: ammonia; N_2_O: nitrous oxide.

We identified differences in emitted N_2_O per kilogram of milk, with a difference of 4.45 g CO_2_ equivalents between rs42786248 homozygotes (Table 3). Considering these genotypes for two hypothetical farms, where animals on the first farm are exclusively bred cows with genotype AA and on the second farm animals are exclusively bred cows with genotype CC. In the case that both farms annually produce a million litres of milk, the second farm would emit 4.45 tons more CO_2_ equivalent (10^6^ × 4.45 g CO_2_ equivalents) emissions. The total milk production in Slovenia in the year 2022 was approximately 625 kilotons of milk,^42^ which, based on the difference between genotypes, would mean that cows with genotype AA would produce 2781 tons of emissions (which is 4.45 g CO_2_ eq/kg milk multiplied with 625,000 kg of milk and divided with 1000 to obtain tons) less than those with genotype CC. More research is needed to study the impact of the genetic background of N_exi_ considering all SNPs that significantly and simultaneously affect both NH_3_ and N_2_O emissions *via* N_exi_.

We included only indirect emissions of N_2_O during housing and both indirect and direct emissions of N_2_O during storage for N_2_O emission calculation. Milk production also generates other N compound emissions, which were not considered in the calculation, and occur in the same and subsequent stages, such as during fertilisation. Additionally, other greenhouse gas emissions, such as enteric CH_4_ emissions and manure CH_4_ emissions, would be included if we assessed all animal-produced emissions. Marshall et al.^43^ found that selecting animals with breeding values for low urea content in milk is environmentally more efficient. Manzanilla-Pech et al.^19^ investigated the genetic background of CH_4_ emissions and, by calculating CH_4_ production per kilogram of energy-corrected milk, identified regions on the genome associated with the CH_4_ emissions intensity. Further research is needed to identify genomic regions associated with the emission intensity of both greenhouse gases, namely CH_4_ and N_2_O. This is essential to incorporate the selection of animals optimally efficient in terms of emissions of both greenhouse gases into breeding programs for environmentally efficient milk production.

## 4. Conclusions

This study provides evidence of the influence of genetic background on N_exi_ in dairy cows. The identified SNPs and genes emerge as potential targets for selective breeding programs aimed at reducing N_exi_ and associated NH_3_ and N_2_O emissions. Genotype AA of rs42786248 SNP within the *MAN1A1* gene is a favourable genotype for the selection of animals aiming for higher milk production with reduced N_exi_. This study contributes to the broader goal of promoting environmental sustainability in milk production, offering a pathway for the sector to address the pressing issue of nitrogen pollution.

## Supplementary Material

SupplementaryTable1.xlsx

Supplementary Figures.docx

## Data Availability

The data that support the findings of this study are available from the corresponding author [DJS] upon reasonable request.
