# Supplementary material for: Identification of genomic regions affecting nitrogen excretion intensity in Brown Swiss dairy cows
Source: Anim Biotechnol. 2024 Dec 10;35(1):2434097. doi: 10.1080/10495398.2024.2434097 (PMC12674215; doi:10.1080/10495398.2024.2434097)
Supplement: Supplementary Figures.docx [file LABT_A_2434097_SM8566.docx]

Identification of genomic regions affecting nitrogen excretion intensity in Brown Swiss dairy cows

Žan Pečnik^1,2^ and Daša Jevšinek Skok ^1*^

^1^Agricultural Institute of Slovenia, Hacquetova ulica 17, 1000 Ljubljana

^2^University of Ljubljana, Kongresni trg 12, 1000 Ljubljana

Supplementary Figures


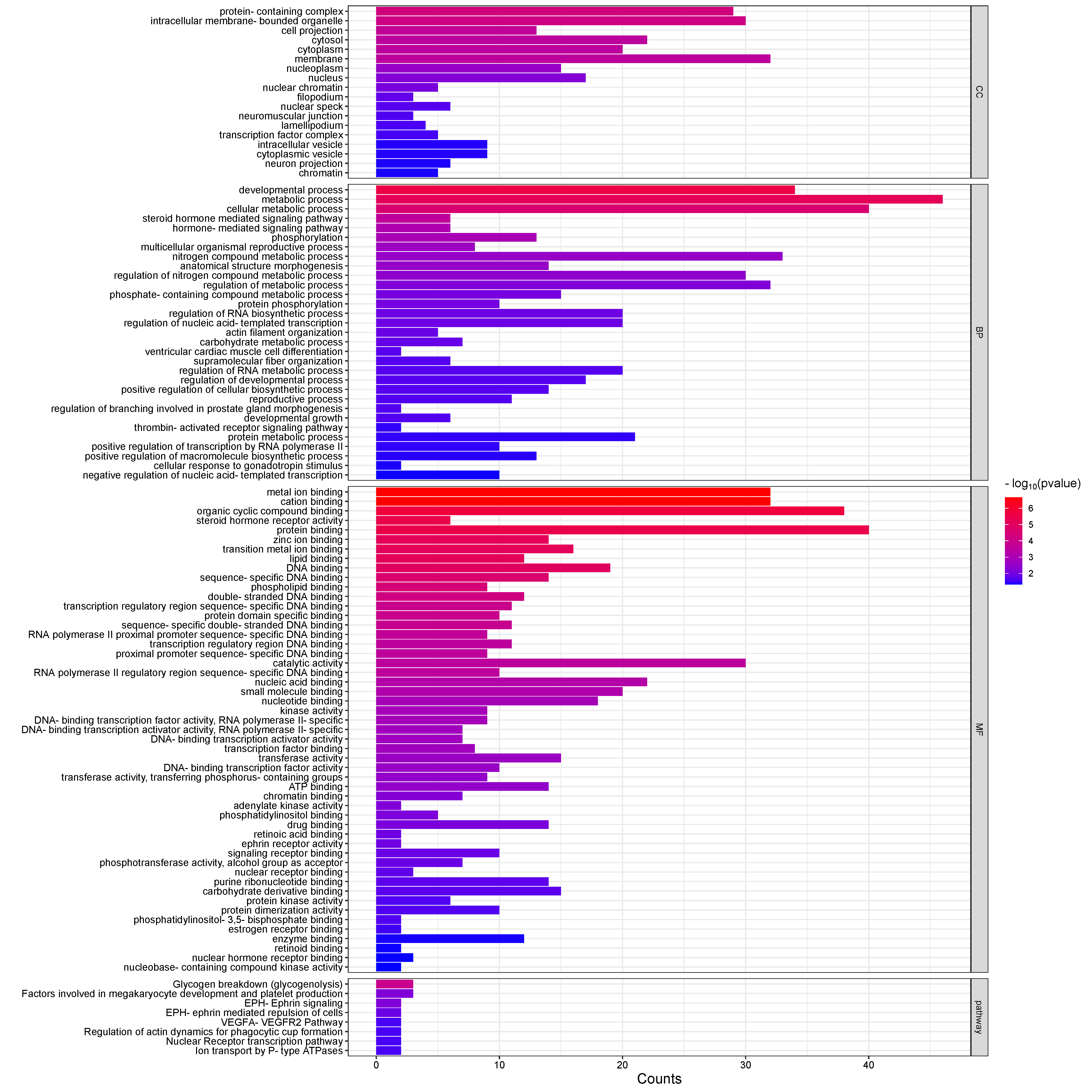
Supplementary Figure 1: All significant Biological Processes (BP), Cellular Components (CC), Molecular Functions (MF) and Pathways based on p-value, identified in functional analysis. Gene ontologies in individual sets are ordered by P/FDR from smaller to larger values.
